# Fish Intake in Pregnancy and Offspring Metabolic Parameters at Age 9–16—Does Gestational Diabetes Modify the Risk?

**DOI:** 10.3390/nu10101534

**Published:** 2018-10-17

**Authors:** Ekaterina Maslova, Susanne Hansen, Marin Strøm, Thorhallur I. Halldorsson, Louise G. Grunnet, Allan A. Vaag, Sjurdur F. Olsen

**Affiliations:** 1 Centre for Fetal Programming, Department of Epidemiology Research, Statens Serum Institut, DK-2300 Copenhagen, Denmark; Susanne.Hansen.10@regionh.dk (S.H.); mrm@ssi.dk (M.S.); lur@ssi.dk (T.I.H.); sfo@ssi.dk (S.F.O.); 2 Department of Primary Care and Public Health, Imperial College, London W6 6RP, UK; 3 Danish Diabetes Academy, DK-5000 Odense, Denmark; louise.groth.grunnet@regionh.dk; 4 Research Centre for Prevention and Health, Rigshospitalet-Glostrup, DK-2600 Copenhagen, Denmark; 5 Faculty of Natural and Health Sciences, University of Faroe Islands, 100 Torshavn, Faroe Islands; 6 Faculty of Food Science and Nutrition, School of Health Sciences, University of Iceland, 101 Reykjavik, Iceland; 7 Unit for Nutrition Research, Landspitali University Hospital, IS-101 Reykjavik, Iceland; 8 Department of Endocrinology-Diabetes and Metabolism, Rigshospitalet, DK-2100 Copenhagen, Denmark; allan.vaag@astrazeneca.com; 9 Cardiovascular and Metabolic Disease (CVMD) Translational Medicine Unit, Early Clinical Development, IMED Biotech Unit, AstraZeneca, 431 50 Gothenburg, Sweden; 10 Department of Nutrition, Harvard T.H. Chan School of Public Health, Boston, MA 02115, USA

**Keywords:** pregnancy, cohort, fish, adiposity, HOMA-IR, GDM

## Abstract

Oily fish, an important source of marine n-3 long-chain polyunsaturated fatty acids (LCPUFA), has shown to reduce cardiometabolic risk in adults. Whether maternal fish intake affects offspring metabolic health is less established, especially among high-risk pregnancies. We aimed to examine the association of fish intake in pregnancy with offspring metabolic health who were either exposed or unexposed to gestational diabetes mellitus (GDM). Our study included 1234 mother-offspring dyads (608 with a GDM index pregnancy and 626 control dyads) nested within the Danish National Birth Cohort, which is a prebirth cohort. Maternal seafood and marine n-3 LCPUFA consumption was quantified by a food frequency questionnaire (gestational week 25) and a sub-sample with interview data (weeks 12 and 30). The offspring were clinically examined at 9–16 years, including a Dual energy X-ray Absorptiometry (DXA) scan and a fasting blood sample. We calculated multivariable effect estimates and 95% confidence intervals (CI) for anthropometric, adiposity, and metabolic parameters. The median (IQR) intake of total seafood was 23(24) g/day. We found largely no association for total seafood and marine n-3 LCPUFA with offspring metabolic parameters in either group. Using interview data, GDM-exposed women reporting no fish in week 12 and 30 (versus intake >2 times/week) had offspring with a higher Body Mass Index (BMI) (ratio of geometric means (RGM): 1.28, 95% CI: 1.06, 1.55), waist circumference (RGM: 1.22, 95% CI: 1.05, 1.40), triglycerides (RGM: 1.77, 95% CI: 1.03, 3.03), and homeostatic model assessment of insulin resistance HOMA-IR (RGM: 2.16, 95% CI: 1.17, 3.97). We found no associations of n-3 LCPUFA and seafood intake with offspring metabolic outcomes. However, GDM-exposed women who consistently reported eating no fish had offspring with a poorer metabolic profile. Fish intake in pregnancy may mitigate some adverse effects of intrauterine hyperglycemia, however, these findings need replication in better powered studies.

## 1. Introduction

Rates of child overweight and obesity have been increasing worldwide, and while they have stabilized in some countries, others remain in an upward trajectory [1]. In Denmark, 8–10% of adolescents aged 11–15 years are reportedly overweight, comparing favorably to most European countries and the US, where the highest prevalence was 31.7% and 25.6% for US boys and girls, respectively [1]. Excess adiposity puts children at risk for impaired glucose tolerance, metabolic syndrome [2], and later, development of type 2 diabetes (T2D) [3,4]. The growing prevalence of overweight/obesity and the associated metabolic risk factors among children and the tracking of these risk factors into adulthood have provoked a search of their early life determinants. Identification of such determinants could assist in implementing more targeted preventive approaches.

Fish, especially oily fish, is a source of marine n-3 long-chain polyunsaturated fatty acids (LCPUFA) (e.g., docosahexaenoic acid, DHA and eicosapentaenoic acid, EPA) that have been associated with a reduced risk of cardiometabolic outcomes in adults [5]. In vitro studies have found that n-3 LCPUFAs may counteract differentiation of preadipocytes into adipocytes, increase expression of anorexigenic hypothalamic peptides, and act on liver transcription factors involved in fatty acid metabolism and gluconeogenesis (reviewed in [6,7]). In animal studies, offspring to dams fed diets supplemented with fish oil or n-3 LCPUFA showed less adiposity [8,9,10,11], lower insulin resistance [9,10,11,12], and reduced cholesterol and triglyceride levels [10] compared to a saturated fat/n-6 LCPUFA diet. In offspring born to diabetic rats, n-3 LCPUFA intake during gestation reduced offspring macrosomia, glucose level, and lipid metabolism [13,14]; however, long-term effects were not examined.

Observational data on fish intake in humans have found that consumption in pregnancy was related to reduced risk of offspring obesity and Body Mass Index (BMI) *z*-scores, though not better proxies of adiposity like skinfolds [15,16]. However, maternal fish intake in late pregnancy was not related to fat mass in two older cohorts examining the offspring at 16 and 30 years [17,18]. More recently, a pooled study of 15 European and US cohorts found that maternal fish intake >3 times/week increased the odds of offspring overweight/obesity at age 4 [19]. While studies using biomarkers of n-3 LCPUFA have found mixed results [15,20,21,22,23,24,25,26,27,28,29,30], several reviews/meta-analyses of randomized clinical trials concluded that there was little support for an effect of n-3 LCPUFA supplementation pre- or perinatally on infant and offspring body composition measures [6,31,32]. However, these were mostly small trials (*n* < 400) that used different disease measures and formulations of n-3 LCPUFA, and administered the intervention at variable time intervals (pregnancy, perinatally, postpartum), making it difficult to make inference from the combined data points and providing a rationale for large observational studies. Furthermore, little is understood about the effect of fish and n-3 LCPUFA intake in high-susceptibility groups like offspring exposed to gestational diabetes mellitus (GDM), despite existing data on the insulin sensitizing effects of n-3 LCPUFA [33] that could potentially reduce offspring exposure to hyperglycemia. This is especially relevant considering altered LCPUFA metabolism in women affected by GDM that influences fetal LCPUFA levels [34,35,36] and demonstrable metabolic ramifications of GDM in these offspring that include greater risk of T2D, obesity, and metabolic syndrome [37,38].

The aim of this study was to examine, in an observational setting, the association of maternal fish intake in pregnancy with offspring markers of metabolic health in GDM-exposed mother-offspring dyads compared to dyads unexposed to GDM. To try and identify a potential etiological agent we also examined marine n-3 LCPUFA intake in a secondary analysis. We hypothesized that offspring exposed to GDM would derive greater metabolic benefits from maternal fish and marine n-3 LCPUFA intake compared to offspring not exposed to GDM.

## 2. Materials and Methods

### 2.1. Study Population

The study population was nested within the Danish National Birth Cohort (DNBC), a longitudinal prospective study with 101,045 pregnancies enrolled between 1996 and 2002 [39]. Participants were interviewed via telephone in gestational week (GW) 12 and 30; and 6 and 18 months postpartum. A food-frequency questionnaire (FFQ) was administered in GW 25. We followed up the mothers and offspring with questionnaires when the offspring turned 7, 11, and 14 years.

**Figure 1:**
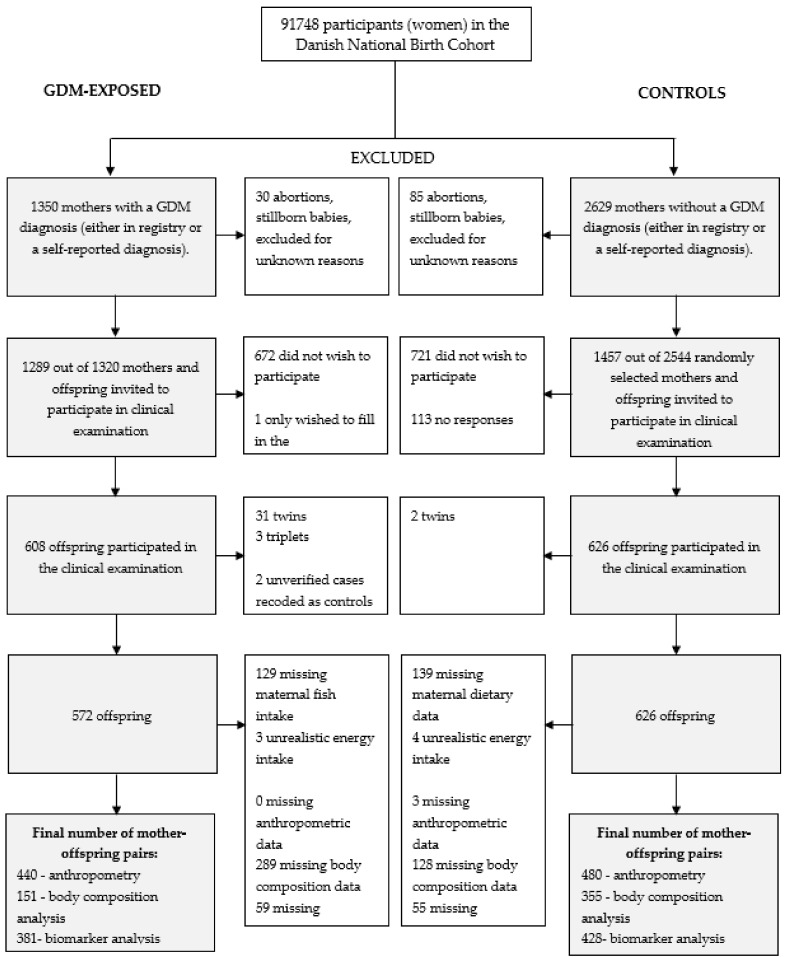
Study population flow (Each woman was defined as either a case or a control in all her pregnancies, thus, redefining some control pregnancies to case pregnancies. A woman who participated with more than one pregnancy was defined as a case if she had had at least one case pregnancy, regardless if the case pregnancy was the first one or not. Initially, there were approximately 50 women with pregnancies defined as both cases and controls; these pregnancies were redefined as cases. Women who only had pregnancies that ended with abortions or stillborn babies were excluded. Some women with live born children were also excluded for an unknown reason. Later in this stage, 470 extra control women were included. GDM: gestational diabetes mellitus).

We recruited offspring born from GDM and non-GDM pregnancies as part of a feasibility study of the offspring born from women enrolled in the Diabetes & Women’s Health (DWH) study nested within the DNBC [40]. This study aimed to study long-term health implications of impaired glucose tolerance in pregnancy on both women and their offspring. We identified 1350 women with suspected GDM using responses to interview questions about a diagnosis in GW 30 and 6 months postpartum and from GDM diagnoses recorded in the Danish National Patient Register (NPR) (ICD-10 codes: O244 and O249) [40,41] (Figure 1); overlap of diagnosis from the two sources was high (positive predictive value: 65% treating NPR as the gold standard). From 91,748 DNBC women, 2629 women (2%) were randomly selected as non-GDM controls. The first phase of GDM case and control identification and the validation of the GDM diagnosis has been elaborated on elsewhere [41]; an additional 470 controls were added in a second phase, which is the number used here. Care was taken to choose controls in the same residential area as the GDM-exposed women and who had had their medical record reviewed by a clinician, although this was not always possible. Medical records were retrieved and reviewed by three clinicians to verify the GDM diagnosis. The reviewers used the World Health Organization (WHO) [42] or published cut-offs for a 2 or 3 h oral glucose tolerance test (OGTT) ([43], Table S1), depending on hospital practice, and took into account physician’s comments that would suggest GDM.

In 2012–2014, we asked the identified GDM-exposed and control mothers and their offspring to participate in a clinical follow-up examination. Of the mother-offspring pairs, 608 (47%) GDM pairs and 626 (43%) controls pairs agreed to the examination (Figure 1). We reassigned two of the GDM-exposed offspring as controls as the women’s GDM diagnoses were listed as ‘unverified’ by the clinical reviewers. Furthermore, we excluded 33 twins (31 GDM) and three triplets (all GDM) to avoid correlated measures due to a shared intrauterine environment. However, to preserve statistical power, we decided to retain 80 siblings in the analysis. Among the 1198 remaining participants, 926 (443 GDM-exposed) had information on maternal total seafood intake, among whom 506 (151 GDM-exposed) had data on body composition.

Mothers participating in the DNBC provided written informed consent for themselves and on behalf of their children. The Regional Scientific Ethics Committee for the municipalities of Copenhagen and Frederiksberg approved all study protocols, and all procedures were in accordance with the Declaration of Helsinki. The clinical follow-up study followed the same procedures with regard to consent and approval of study protocols (H-4-2011-045 and H-4-2013-129).

### 2.2. Maternal Dietary Intake

#### 2.2.1. Food Frequency Questionnaire Data

Maternal diet was evaluated in GW 25 using a 360-item semi-quantitative FFQ that asked about the women’s intake in the past month [44]. We used assumptions of standard portion sizes to calculate nutrient intake. We quantified food intakes in grams per day and nutrient intake was calculated using the National Food Institute’s Food Composition Databank version 6.02 (www.foodcomp.dk). All nutrients were energy-adjusted using the residual method [45]. Pregnant women reporting intake resulting in unrealistic energy intake estimates (arbitrarily set to <2500 kJ/day or >25,000 kJ/day) were removed.

The FFQ has been validated against seven-day weighed food diaries and biomarkers of selected nutrients (*n* = 88) [46]. Intake of n-3 LCPUFA quantified using the FFQ was moderately correlated with both erythrocyte EPA (Spearman rho = 0.37) and food diaries (rho = 0.28). Correlations with fish oil supplementation were not examined, however, <3% of women in the DNBC reported taking these supplements [44]. Our primary exposure was total seafood (oily fish, lean fish, shellfish, smoked fish, fish spread) intake (g/day) derived from the FFQ. In secondary analyses, we further subdivided total seafood into lean and oily fish; additionally, we examined the intake of dietary marine n-3 LCPUFAs (g/day).

#### 2.2.2. Interview Data

Additionally, we assessed consistent fish intake using pregnancy interviews in GW 12 and 30, which limited the analysis to women reporting intake at both occasions, thereby reducing measurement error compared to the FFQ data and limiting overlap between intake categories. We used a definition of fish intake developed for the DNBC that best differentiated among the levels of intake. This definition evaluated fish intake with a sandwich or a hot meal, the most common ways of consuming fish in Denmark, and did not differentiate between species of fish consumed, with five categories of intake: (1) never eating fish according to both interviews; (2) hot meal and sandwiches both eaten monthly or less than each month, according to both interviews; (3) hot meal monthly and sandwiches weekly, according to both interviews; (4) sandwiches and hot meals both eaten weekly according to both interviews, at a low frequency (hot meals 1 time/week and sandwiches 1–2 times/week, in at least one interview); (5) sandwiches and hot meals both eaten weekly at a high frequency (hot meals >2 times/week or sandwiches >3 times/week, according to both interviews). The last group was used as the reference due to the small sample size in group 1 and because there was an interest to determine the risk of consistently never consuming fish in pregnancy.

### 2.3. Offspring Follow-Up

A single clinical examination of the offspring that included anthropometry, body composition, and a fasting blood sample was conducted at age 9–16 years. Research or student assistants/nurses (RA) measured height and weight in offspring when they were lightly dressed and without shoes using a Charder measuring device (Charder Electronic Coltd, Taichung City, Taiwan) and a Rex BWB-800 MA scale (Tanita Corporation, Tokyo, Japan), respectively. BMI was calculated as kg/m^2^. Waist circumference was measured at the naval using a soft tape while standing. All measurements were taken twice and a difference of >0.5 cm resulted in a third measurement. An average of all available measurements was calculated (either two or three measurements).

Body composition was assessed in a subset of the offspring with a Dual energy X-ray Absorptiometry (DXA) scan (ProdigyDEXA GE Lunar Prodigy, Madison, WI, USA). We used total and abdominal fat percentage as measures of overall and central adiposity.

We assessed blood glucose and lipid (total cholesterol, low-density lipoproteins (LDL) and high-density lipoproteins (HDL)) concentrations (mmol/L) employing standard laboratory methods on the Modular P-module (Roche, Mannheim, Germany). A sandwich electrochemiluminescence immunoassay was used to quantify fasting plasma insulin levels (pmol/L) (Roche Diagnostics, Basel, Switzerland). All technicians were blinded to whether offspring were GDM-exposed or controls. We calculated homeostatic model assessment of insulin resistance (HOMA-IR) as ([(fasting plasma insulin × fasting plasma glucose)/22.5] × 0.144) [47].

To evaluate overall cardiometabolic health, we constructed a continuous metabolic syndrome (MetS) *z* score using offspring BMI, waist circumference, fasting blood glucose, fasting insulin, triglycerides (TG), HDL, and systolic blood pressure z-scores. Component inclusion was based on a validation study of the MetS score against a future T2D diagnosis in Pima Indians [48]. In the GDM-exposed offspring, the cardiometabolic components were weighted by hazard ratios taken from the association of each component with a T2D diagnosis; diastolic blood pressure was left out because it was not associated with T2D (Table 3, ages 10–14 years in [48]). We used weights for GDM-exposed offspring as both Pima Indians and individuals exposed to intrauterine hyperglycemia are considered high-risk populations that are at an increased risk of overweight/obesity and T2D compared to the general population [37,38,49,50,51]. Scores were kept unweighted in the control offspring, as they have a different risk profile and because, to our knowledge, no validated weights were available for low-risk populations. A higher MetS *z* score indicated poorer cardiometabolic health.

The outcomes examined in this study included BMI, waist circumference, total and abdominal adiposity, cholesterol (total, LDL, HDL), tryglycerides, HOMA-IR, and MetS *z* score. BMI and waist circumference were examined as absolute values and age- and sex-specific *z* scores based on the full study population. As the results were similar, we present only the results for the former. Since our interest was in the overall metabolic profile, we did not specify primary and secondary outcomes, nor adjust for multiple comparisons. Instead, we evaluated the results based on consistency and strengths of association for the purpose of generating further hypotheses in the examined sub-populations.

### 2.4. Covariates

We included model covariates previously known to be associated with offspring metabolic outcomes [17,52,53,54,55]. All maternal/parental covariates apart from smoking were derived from interview 1 (GW 12) data; smoking used both interview 1 and 2 data. Offspring covariates were collected as part of the DWH study. In our model we adjusted for: maternal age (continuous, 0.1% missing data), parental socioeconomic position (based on parental occupation and education: high level position, medium level position, skilled, unskilled, and unemployed/student, 3.7% missing data), parity (nulliparous, 1 prior child, ≥2 prior children, 3.2% missing data), maternal prepregnancy BMI (≤18.5, 18.6–24.9, 25.0–29.9, 30.0–34.9, ≥35 kg/m2, 4.6% missing data), maternal smoking in pregnancy (nonsmoker, occasional smoker, smoker, 0.5% missing data), total maternal physical activity based on structured and unstructured activities regardless of level (none, 1–119 min/week, ≥120 min/week, 4.0% missing data), and offspring age (continuous, 0.0% missing data) and sex (male, female, 0.0% missing data). We used a missing indicator to account for missing data >1%; data missing ≤1% was deleted. A complete case analysis was run as a sensitivity analysis.

In a secondary analysis, we also adjusted for breastfeeding duration and the offspring’s own fish intake at age 14 years that included fatty fish, lean fish, shellfish, and fish cold cuts. Waist circumferences and MetS *z* score was furthermore adjusted for offspring height [56,57].

### 2.5. Statistical Analysis

We conducted all analyses separately for GDM-exposed and control mother-offspring dyads. In the descriptive analysis, we calculated mean (SD) for continuous variables and frequencies (%) for categorical maternal variables. We examined selected maternal dietary intakes and covariates across categories of total seafood intake. F-test and Chi-square test were used to calculate statistical significance for continuous and categorical variables, respectively, accounting for correlated values among siblings. We used Spearman correlations (rho) to quantify the relation between maternal total seafood intake in pregnancy and that of the offspring at the 14 years’ follow-up.

We considered maternal dietary exposures as categorical to assess potential non-linear associations. Total seafood and fish intake categorization was based on variable data structure and current recommendations by the U.S Food and Drug Administration (FDA) and the Environmental Protection Agency (EPA) of 8–12 oz of fish per week in pregnancy, with the higher category roughly equal to the FDA-EPA recommendation [58]; marine n-3 LCPUFA intake was categorized into quartiles. We calculated *p*-values evaluating the null hypothesis of no differences across categorical effect estimates. To allow for the lack of independence among sibling observations (6.6% of total population), we used multivariable linear mixed regression models with a compound symmetry covariance structure to calculate mean differences and 95% confidence interval (CI). Model residuals were examined using quantile-quantile (Q-Q) plots for all linear regressions to assess normality. If outcomes were found not to be normally distributed, they were log-transformed; the effect estimates and 95% CI were exponentiated and reported as ratios of geometric means (RGM) with a null value of 1. This translates into a (RGM-1) percentile change in the outcome for direct associations, and vice versa for inverse associations. Adjustment for offspring fish intake at age 14 years was carried out in a secondary analysis to examine the association for maternal fish and n-3 LCPUFA intake independent of the offspring own fish intake. For the primary exposure, we also evaluated interactions with offspring gender using a cross-term between the exposure and gender. If the interaction term was statistically significant (*p* < 0.05) in any of the exposure categories, we stratified on gender.

All tests were two-sided with a threshold of *p* < 0.05 to denote statistical significance. The analyses were performed using the Statistical Analyses System software (release 9.4; SAS Institute, Cary, NC, USA).

## 3. Results

### 3.1. Study Population

The median (IQR) intake of total seafood in GW 25 was 0.8 (0.4–1.3) oz/day in the GDM-exposed and 0.8 (0.5–1.3) in the control women. The median (IQR) intake of marine n-3 LCPUFA was 0.3 (0.2–0.5) g/day in GDM-exposed women; intake was slightly higher in the control (0.4 (0.2–0.6)) women. The correlation between maternal pregnancy and offspring 14-year total seafood intake was 0.21(*p* < 0.0001) and 0.17 (*p*: 0.0001) in GDM-exposed and control dyads, respectively.

Lean fish, oily fish, marine n-3 LCPUFA, and protein intake in GW 25 increased across categories of total seafood intake in both groups (Table 1); additionally, control women in the higher categories had lower carbohydrate intake than those in the lower categories. Among GDM-exposed women, the proportion of those with pre-pregnancy BMI 18.6–24.9 kg/m^2^ increased across the total seafood intake categories, however, no differences were found for any of the other variables in either group. Anthropometric and metabolic differences between control and GDM-exposed offspring have been evaluated in a separate study [59]; sociodemographic differences between GDM-exposed and control women has been published previously [60].

We compared GDM-exposed women with (*n* = 443) and without (*n* = 129) data on total seafood intake in GW 25. We found that women with total seafood intake data were more likely to be nulliparous (38% vs. 30%), of high-medium sociodemographic position (50% vs. 42%), to be normal weight (40% vs. 30%), and less likely to not exercise (64% vs. 70%). Among the control women, 139 women out of 626 were lacking information on total seafood consumption. There were differences between women with and without dietary information in parity (nulliparity: 51% vs. 45%), sociodemographic position (high/medium: 65% vs. 54%), pre-pregnancy BMI (18.6–24.9 kg/m^2^: 74% vs. 66%), smoking (non-smoker: 79% vs. 71%) and offspring waist circumference (69 vs. 71 cm).

### 3.2. Maternal Fish Intake and Offspring Metabolic Outcomes in the GDM-Exposed Group

There were no associations for total seafood (Table 2), lean (Table S4), or oily (Table S5) fish in GW 25 with any of the offspring outcomes. Statistically significant gender interactions were found for total seafood intake with abdominal fat percentage. Stratifying on gender, the associations with abdominal fat percentage was 1.49 (95% CI: 0.95, 2.32) for males and 1.11 (95% CI: 0.79, 1.52) for females when comparing the highest to lowest category of total seafood intake.

The results for consistent fish intake across pregnancies using frequencies of fish intake as a sandwich or hot meal showed 28% higher BMI (RGM: 1.28; 95% CI: 1.06, 1.55) and 22% higher waist circumference (RGM: 1.22; 95% CI: 1.05, 1.40) in offspring whose mother consistently reported never eating fish in pregnancy compared to a consistently high intake of >2 portions/week (Table 3). Offspring in this group also had higher triglycerides (RGM: 1.77; 95% CI: 1.03, 3.03) and HOMA-IR (RGM: 2.16, 95% CI: 1.17, 3.97), associations that were present already in the 1–2 times/week intake category. This translated into a higher MetS *z* score among these offspring (never vs. high intake >2 portions/week: 12.91, 95% CI: 4.07, 21.75 SD). These associations were only apparent in the ‘never’ group and did not show any indications of a dose-response. The women in the never category were more likely to be older (34.2 vs. 32.7 years), to be primi/multiparous (88% vs. 69%), to never exercise (88% vs. 73%), to be overweight/obese (75% vs. 57%), and more likely to smoke (63% vs. 26%) compared to the rest of the GDM-exposed women. Changing the highest category frequency to >3 times/week consistent with the recently published pooled analysis [19] did not alter the results (data not shown). Further adjusting for breastfeeding duration did not markedly alter the conclusion, although the association between total seafood and TG levels in GDM-exposed offspring became statistically significant (RGM: 0.84, 95% CI: 0.72, 0.98). Convergence problems due to small numbers made it difficult to evaluate the effect of breastfeeding adjustment in the consistent fish intake analysis. Adjustment for offspring seafood intake at age 14 years did not alter the results, while adjusting the waist circumference and MetS *z* score for offspring height somewhat strengthened the effect estimates. A complete case analysis attenuated the effect estimates for consistent fish intake; however, this did not alter the conclusions of the results.

Statistically significant interactions for gender with consistent fish intake from the interviews were present for total and abdominal fat percentage and HDL. The power for adiposity measures was too low for stratification. Comparing never eating fish in pregnancy to a consistently high intake of >2 portions/week, the RGM for HDL was 1.05 (95% CI: 0.53, 2.10) for male offspring and 1.07 (95% CI: 0.59, 1.93) for female offspring.

### 3.3. Maternal Fish Intake and Offspring Metabolic Outcomes in the Control Group

There were no associations of maternal total seafood intake in GW 25 and any of the offspring metabolic outcomes apart from a marginal association with HDL (>1.5 oz/d vs. 0–0.5 oz/d: mean Δ: 0.13; 95% CI: 0.01, 0.24 mmol/L) (Table S2). We found no significant interactions with gender.

There were no significant associations for consistent fish intake assessed by interviews (Table S3). We found significant gender interactions for total cholesterol and LDL levels. Because of low cell numbers, the effect estimates in the ‘never’ category could not be calculated. Comparing the second lowest to the highest category, the RGM for total cholesterol was 0.71 (95% CI: 0.38, 1.32) for male offspring and 1.58 (0.90, 2.77) for female offspring. The effect estimates were similar for LDL, however, these reached statistical significance for female offspring only (RGM: 1.72, 95% CI: 1.00, 2.97). A complete case analysis gave similar results.

In secondary analyses, maternal lean fish intake in GW 25 in the highest category (>0.5 oz/d) was associated with a lower offspring total fat mass percentage (RGM: 0.89, 95% CI: 0.80, 0.97) and abdominal fat mass percentage (RGM: 0.84; 95% CI: 0.70, 0.99) compared to the lowest intake category (0 oz/d) (Table S4). Maternal lean fish intake was also related to higher offspring HOMA-IR (1.22; 95% CI: 1.04, 1.43). We found no significant associations for oily fish in GW 25 (Table S5). Adjusting for breastfeeding duration, offspring cross-sectional seafood intake, or offspring height (waist circumference and MetS models) did not substantially alter the results. Similar convergence issues with the breastfeeding adjustment as noted for the GDM-exposed offspring analysis were present here.

### 3.4. Maternal Dietary n-3 LCPUFA Intake in GW 25 and Offspring Metabolic Outcomes

We found no associations for any of the offspring metabolic outcomes in the GDM-exposed or control group (Table S6).

## 4. Discussion

In this study, we found that the association of maternal fish intake with offspring metabolic profiles differed by maternal hyperglycemia status (i.e., GDM vs. non-GDM) only for consistent fish intake in a subsample of the study population; associations with seafood, lean and fatty fish, and marine n-3 LCPUFA were largely null. Among offspring born from pregnancies complicated by GDM, maternal reports of never eating fish in both early and late pregnancy was related to an overall poorer metabolic profile in the GDM-exposed offspring only, with higher BMI, waist circumference, insulin resistance, and triglyceride levels. No significant associations were observed among offspring born from GDM unexposed pregnancies. There were some suggestions of gender-specific effects; however, the power was generally too low to draw any confident conclusions.

Prior studies have largely focused on n-3 LCPUFA status in pregnancy and fewer studies have examined fish intake in relation to offspring metabolic outcomes; furthermore, data in offspring exposed to GDM is largely lacking. Studies on fish intake in population-based cohorts have found inverse associations with offspring BMI or obesity in the first seven years of life [15,16], though not in adolescence or adulthood [16,17,18], which is consistent with our null results for total seafood intake. Unlike our analysis, none of the prior studies distinguished between lean and oily fish intake and used composite measures of fish intake. Timing of exposure may also play a role; only one study examined fish intake in early pregnancy and found it related to lower offspring percent body fat at age 30 [17]. A recent pooled analysis of 15 European and US cohorts found that fish intake greater than three times a week (vs. ≤1 time/week) was related to higher levels of obesity in children in early childhood [19]. The authors proposed that higher levels of mercury or organic pollutants may account for the findings; however, this hypothesis could not be tested directly. Conversely, we found that maternal lean fish consumption of >0.5 oz/day (roughly equivalent to 1 serving/week) was associated with reduced total and central adiposity among control offspring. Since only 1% (*n* = 5) of the control women reported intake ≥1.3 oz/day or approximately 2 servings/week, we cannot preclude harmful effects at higher intake levels. The lack of associations in the GDM-exposed group with the body composition measures could have depended on the smaller sub-sample of these offspring that agreed to a DXA scan.

None of the prior studies have separately examined the association between maternal fish intake and offspring metabolic health in GDM pregnancies, most likely due to a lack of power, with GDM present in 2–6% of pregnancies in European countries [61] and about 8% in the USA [62]. The present study design oversampled GDM pregnancies to more thoroughly examine differential associations in GDM-exposed vs. unexposed offspring. The main differences were noted for consistent fish and lean fish exposure. Never eating fish in both early and late pregnancy was related to poorer offspring metabolic parameters in the GDM-exposed offspring only. However, only eight women reported intake at this level. These women were more likely to be older, primi/multiparous, to never exercise, to be overweight/obese, and were more likely to smoke compared to the rest of the GDM-exposed women. While experimental work in a rat model of chemically-induced diabetes in pregnancy provide some support to mitigating effects of marine n-3 LCPUFA in offspring exposed to intrauterine hyperglycemia [13,14], definitive conclusions could not be reached in our study due to the small sample size and potential residual confounding. This was more pertinent to the BMI and waist circumference results where significant effect estimates were present only in the ‘never’ group. Conversely, levels of triglycerides and HOMA-IR were increased also in the other fish intake categories, suggesting a threshold effect. We did not find any associations for this exposure in the control group; however, there were only five women in the ‘never’ intake group. These women were on average 29.4 years, primiparous (80%), of normal weight (80%), and non-smoking (80%), in line with Table 1 showing control women to be somewhat younger, more likely to be of normal weight, and smoke less.

Significant associations in the controls were present for total seafood and lean fish intake. We found a direct association between maternal total seafood intake and HDL. The directionality of this association was consistent across lean fish, oily fish, and n-3 LCPUFA intake, although the confidence intervals included the null value. We also found suggestive inverse associations with LDL for total seafood intake; however, these were not statistically significant. These findings are overall consistent with the prior evidence on metabolic biomarkers, which has examined n-3 LCPUFA status rather than fish intake. An intervention study in a different Danish population found that fish oil supplementation in pregnancy reduced triglycerides and cholesterol and increased HDL in 19–21 years old offspring, though these were not statistically significant [27]. In another Danish cohort of 965 women with largely comparable intake levels, marine n-3 LCPUFA intake was not related to lipid levels [25]. This is in contrast to a recent study using maternal plasma levels of fatty acids which found that DHA levels were associated with higher offspring cholesterol and HDL at an age of six years [63]. Interestingly, this study also found that maternal DHA plasma levels were related to higher offspring insulin. We found similar results with HOMA-IR and fasting insulin (data not shown), though only for lean fish intake, which is a poorer source of n-3 LCPUFA compared to oily fish. These associations were strengthened by adjusting for total offspring adiposity (data not shown), implying that nutritional or non-nutritional components in lean fish may act on insulin resistance capabilities independently of adiposity. This was in contrast to past studies reporting either inverse results for short-term insulin levels [28,64] or null findings for studies with longer follow-up [24,25,65]. Our results were also contradictory to the inverse results we found for lean fish intake with adiposity, pointing to either a chance finding or differential etiology for glucose and lipid homeostasis, something that we could not determine using these data. Similar associations were not present in the GDM-exposed offspring, suggesting that lean fish may have differential interactions with hyperglycemia and concomitant excess adiposity in pregnancy. However, we cannot preclude design or analytical limitations, including larger exposure measurement error in the GDM-exposed group due to overlapping timing of GDM diagnosis and FFQ administration, or chance findings due to multiple testing.

Dietary data on marine n-3 LCPUFA remain equally conflicting with both inverse [15] and null [25,66,67] associations reported in the literature. Our findings in both groups are largely in line with the null results from these studies. Studies where the mean/median marine n-3 LCPUFA intake was reported or could be estimated, intake levels were generally higher in the null studies. This suggests that exposure dose cannot explain the null findings and neither could the timing of the outcome assessment, which varied between birth and 19 years. However, given the small intake amounts of marine n-3 LCPUFA, measurement error may be more pronounced for intake of LCPUFA compared to fish intake. It is also possible that it is the combined nutrients and nutritional synergies provided by fish that matter for optimal development of metabolic pathways and organ systems rather than n-3 LCPUFA alone. This is supported by both biomarker studies and interventions of n-3 LCPUFA in pregnancy that have generally found no effect on either BMI or adiposity measures [68]. Other nutrients in fish, e.g., vitamin D, may account for the beneficial associations observed in this analysis and this has been supported by recent observational studies [69,70,71]. Additionally, it is plausible that fish meals may be substituting less healthy options in pregnant women’s diets, however, this needs to be more rigorously examined in a food substitution analysis.

The main limitation of the study was the overlap in time between dietary exposure assessment and GDM diagnosis. This could have led to misreporting of intake due to either real or perceived changes in diet that may not have reflected intake prior to diagnosis. Data on the date of diagnosis was not available for the majority of women and could not be accounted for in the analysis. Furthermore, we lacked reliable data on GDM treatment and were not able to consider this in the analysis. Fish intake may have different effect on offspring metabolic health depending on the level of maternal glucose control, and this interaction is worth assessing in studies with available data on treatment patterns. Fish intake assessed in interviews had the advantage of being assessed before diagnosis and women would have only been included if their intake was consistent post-diagnosis. The interview variable also appeared better at separating out high and low consumers compared to the FFQ measures of fish and LCPUFA intake and could have precluded finding associations with these exposures. The marine n-3 LCPUFA range in the lowest total seafood was 0.05–0.95 g/day intake category which overlapped almost completely with the highest category (range 0.10–2.76 g/day). The overlap was smaller in the interview categories where the respective numbers were 0.08–0.54 g/day (range in lowest category) and 0.21–2.28 g/day (range in highest category). For the consistent fish intake, only mothers who had information on fish intake at both interviews were included and the sample size was, therefore, cut down to about a third of the study population. The interviews did not capture information on preparation method or context in which the fish meal or sandwich was consumed (out or at home), which would have affected the meal’s nutrient composition. Importantly, we found a beneficial association for high consistent fish intake with offspring metabolic parameters regardless of these factors. GDM-exposed women who did have complete fish interview data were generally of higher sociodemographic status with lower BMI and were less likely to smoke compared to those without data; however, there were no substantial differences in the outcome measures to suggest a significant selection bias (data not shown). The sample size was particularly cut down in the ‘never’ fish eating group (*n* = 8) where women were more likely to be overweight/obese and to smoke, which could have led to confounding that was difficult to fully adjust for. We cannot therefore exclude the potential for residual or even intractable confounding, particularly for the BMI and waist circumference findings. For the remaining dietary variables, the minor changes between the unadjusted and adjusted effect estimates suggest that even some potentially strong confounders like maternal BMI had only minor influence on the results. The results of the present study should be considered in light of changes that have occurred since this cohort was enrolled. Following global trends, the prevalence of GDM in Denmark is increasing, though it is still quite low on an absolute level (2.9% in 2012 vs. 1.7% in 2004), which is consistent with the low GDM prevalence in this cohort (1.3%) [72,73]. This may suggest that the findings in the present study may be even stronger in today’s population. However, this likely depends on the level of glucose control in pregnancy on which we did not have reliable data. Generalizability may also depend on current use of n-3 LCPUFA supplements; however, levels of fish oil use was low in our cohort (2.2%) and the Danish Health Authorities currently do not recommend fish oil supplements to pregnant women [74]. Another important effect modifier may be maternal adiposity; however, teasing this apart from GDM would require a study design specifically aimed at examining this issue with sufficient power.

The strengths of this study include its longitudinal, prospective design that allowed us to examine long-term implications for maternal fish and marine n-3 LCPUFA intake in pregnancy on offspring metabolic health in late childhood and adolescence. This was done separately for GDM-exposed and control mother-offspring dyads because of oversampling of the former group, allowing for the study of fish intake in this more metabolically vulnerable sub-group. We had detailed dietary data that included fish intake assessed in both early, mid, and late pregnancy. The offspring outcomes were measured during a clinical visit by RAs unaware of the maternal fish intake and were therefore not subject to reporting bias. The DNBC also collected extensive data on many prenatal and postnatal confounders that were considered for the multivariable model to limit residual confounding.

To conclude, we found no associations of marine n-3 LCPUFA and seafood intake with offspring metabolic outcomes. However, GDM-exposed women who consistently reporting eating no fish in pregnancy had offspring with a poorer metabolic profile. Maternal fish intake as a potential mitigator of offspring metabolic health outcomes among high-risk offspring needs to be more thoroughly examined in studies of larger sample size and longer term of follow-up.

## Figures and Tables

**Table 1:** Maternal characteristics distributions across quartiles of total seafood intake in gestational week 25 across gestational diabetes mellitus (GDM)-exposed and control women.

**Categories of Total Seafood Intake**					
	0–0.5 oz/day	>0.5–1 oz/day	1–1.5 oz/day	>1.5 oz/day	*p* ^1^
GDM-exposed (*n* = 443)	% or means (SD)	% or means (SD)	% or means (SD)	% or means (SD)	
Lean fish intake (oz/day)	0.1 (0.1)	0.2 (0.2)	0.4 (0.2)	0.6 (0.4)	<0.0001
Oily fish intake (oz/day)	0.0 (0.1)	0.1 (0.1)	0.2 (0.2)	0.3 (0.3)	<0.0001
Marine n-3 LCPUFA (g/day)	0.2 (0.1)	0.4 (0.2)	0.5 (0.2)	0.8 (0.5)	<0.0001
Total fat intake (g/day)	83.4 (18.2)	81.9 (15.8)	79.5 (16.1)	79.2 (16.0)	0.20
Carbohydrate intake (g/day)	317.4 (36.9)	313.6 (38.3)	317.8 (35.3)	309.8 (35.4)	0.33
Protein intake (g/day)	88.3 (15.8)	91.7 (12.4)	95.5 (13.6)	102.7 (15.7)	<0.0001
Maternal age (years)	31.2 (4.6)	32.1 (4.2)	32.4 (4.1)	32.2 (4.5)	0.12
Nulliparous (%)	44	36	38	29	0.32
High/medium sociodemographic position (%)	49	45	56	57	0.63
No physical activity (min/week) (%)	65	60	67	68	0.51
Pre-pregnancy BMI 18.6–24.9 kg/m^2^ (%)	35	41	42	46	0.05
Gestational weight gain (kg/week)	0.4 (0.4)	0.4 (0.3)	0.4 (0.3)	0.4 (0.4)	0.72
Non-smokers in pregnancy (%)	71	70	71	78	0.78
Breastfeeding ≥10 months (%)	17	21	33	28	0.34
Birth weight (g)	3786 (623)	3709 (719)	3766 (471)	3801 (590)	0.75
Gestational age (days)	277 (11)	276 (11)	278 (11)	278 (12)	0.57
**Categories of Total Seafood Intake**					
	0–0.5 oz/day	>0.5–1 oz/day	1–1.5 oz/day	>1.5 oz/day	*p* ^1^
Controls (*n* = 487)	% or means (SD)	% or means (SD)	% or means (SD)	% or means (SD)	
Lean fish intake (oz/day)	0.1 (0.1)	0.3 (0.2)	0.4 (0.2)	0.6 (0.4)	<0.0001
Oily fish intake (oz/day)	0.0 (0.1)	0.1 (0.1)	0.2 (0.2)	0.4 (0.3)	<0.0001
Marine n-3 LCPUFA (g/day)	0.2 (0.1)	0.4 (0.2)	0.5 (0.3)	0.8 (0.4)	<0.0001
Total fat intake (g/day)	78.1 (16.0)	80.0 (16.0)	79.3 (14.9)	78.8 (12.8)	0.75
Carbohydrate intake (g/day)	330.2 (34.1)	319.8 (36.5)	318.2 (32.9)	316.6 (30.0)	0.01
Protein intake (g/day)	84.7 (13.8)	90.0 (12.4)	93.3 (12.9)	94.9 (13.7)	<0.0001
Maternal age (years)	30.2 (3.8)	31.2 (4.1)	31.7 (4.4)	31.7 (4.2)	0.07
Nulliparous (%)	54	52	49	47	0.82
High/medium sociodemographic position (%)	63	60	70	69	0.10
No physical activity (min/week) (%)	59	60	60	56	0.94
Pre-pregnancy BMI 18.6–24.9 kg/m^2^ (%)	67	74	74	81	0.35
Gestational weight gain (kg/week)	0.5 (0.2)	0.5 (0.2)	0.5 (0.2)	0.5 (0.2)	0.92
Non-smokers in pregnancy (%)	77	78	79	81	0.70
Breastfeeding ≥10 months (%)	29	31	21	34	0.17
Birth weight (g)	3619 (473)	3587 (482)	3594 (551)	3537 (519)	0.65
Gestational age (days)	281 (10)	281 (10)	281 (13)	280 (11)	0.82

1 oz = 28.35 g. BMI: body mass index; GDM: gestational diabetes mellitus; LCPUFA: long-chain polyunsaturated fatty acids; SD: standard deviation.^1^ Mean (SD) displayed for continuous variables and % for categorical variables. Statistically significant differences across intake categories were determined using F-test or Chi-square test for continuous and categorical variables, respectively. All *p*-values were corrected for correlations among siblings.

**Table 2:** The association between total seafood intake in gestational week 25 and offspring metabolic parameters at age 9–16 years in GDM-exposed mother-offspring dyads.

Categories of Total Seafood Intake					
	0–0.5 oz/day	>0.5–1 oz/day	1–1.5 oz/day	>1.5 oz/day	*p* ^1^
Metabolic Measures in the Offspring	*n* = 142 (32%)	*n* = 136 (31%)	*n* = 96 (22%)	*n* = 69 (16%)	
BMI, kg/m^2^ (*n* = 440)					
Unadjusted RGM (95% CI)	1 (ref)	0.98 (0.94, 1.02)	0.97 (0.93, 1.02)	0.99 (0.94, 1.04)	0.69
Adjusted RGM ^2^ (95% CI)	1 (ref)	0.98 (0.94, 1.02)	0.99 (0.94, 1.03)	1.01 (0.96, 1.05)	0.65
Waist circumference, cm (*n* = 438)					
Unadjusted RGM (95% CI)	1 (ref)	0.99 (0.96, 1.03)	0.97 (0.93, 1.01)	1.00 (0.95, 1.04)	0.39
Adjusted RGM ^2^ (95% CI)	1 (ref)	0.99 (0.96, 1.02)	0.98 (0.94, 1.01)	1.00 (0.96, 1.04)	0.49
Total fat mass, % (*n* = 151)					
Unadjusted RGM (95% CI)	1 (ref)	1.04 (0.93, 1.17)	0.99 (0.87, 1.13)	1.07 (0.92, 1.25)	0.68
Adjusted RGM ^2^ (95% CI)	1 (ref)	1.07 (0.96, 1.20)	0.95 (0.84, 1.07)	1.12 (0.96, 1.28)	0.12
Abdominal fat mass, % (*n* = 151)					
Unadjusted RGM (95% CI)	1 (ref)	1.09 (0.88, 1.36)	0.97 (0.76, 1.22)	1.16 (0.88, 1.54)	0.52
Adjusted RGM ^2^ (95% CI)	1 (ref)	1.12 (0.91, 1.38)	0.93 (0.75, 1.17)	1.26 (0.96, 1.63)	0.13
Total cholesterol, mmol/L (*n* = 381)					
Unadjusted mean Δ (95% CI)	0 (ref)	−0.05 (−0.23, 0.14)	0.09 (−0.11, 0.29)	−0.13 (−0.36, 0.10)	0.31
Adjusted mean Δ^2^ (95% CI)	0 (ref)	−0.01 (−0.19, 0.17)	0.00 (−0.20, 0.20)	−0.09 (−0.31, 0.13)	0.86
LDL, mmol/L (*n* = 381)					
Unadjusted mean Δ (95% CI)	0 (ref)	−0.01 (−0.18, 0.16)	0.09 (−0.09, 0.28)	−0.15 (−0.36, 0.06)	0.20
Adjusted mean Δ^2^ (95% CI)	0 (ref)	0.02 (−0.15, 0.19)	0.02 (−0.16, 0.21)	−0.10 (−0.30, 0.10)	0.65
HDL, mmol/L (*n* = 381)					
Unadjusted mean Δ (95% CI)	0 (ref)	−0.03 (−0.12, 0.06)	−0.02 (−0.12, 0.08)	0.05 (−0.06, 0.17)	0.52
Adjusted mean Δ^2^ (95% CI)	0 (ref)	−0.03 (−0.13, 0.06)	−0.05 (−0.16, 0.05)	0.04 (−0.07, 0.16)	0.43
TG, mmol/L (*n* = 381)					
Unadjusted RGM (95% CI)	1 (ref)	0.93 (0.84, 1.03)	0.95 (0.84, 1.06)	0.85 (0.75, 0.97)	0.11
Adjusted RGM ^2^ (95% CI)	1 (ref)	0.92 (0.83, 1.03)	0.96 (0.86, 1.08)	0.89 (0.78, 1.01)	0.26
HOMA-IR (*n* = 361)					
Unadjusted RGM (95% CI)	1 (ref)	0.99 (0.86, 1.15)	0.91 (0.77, 1.07)	1.00 (0.83, 1.20)	0.70
Adjusted RGM ^2^ (95% CI)	1 (ref)	1.00 (0.87, 1.16)	0.97 (0.83, 1.14)	1.02 (0.85, 1.22)	0.97
MetS ***z*** score, SD (*n* = 359)					
Unadjusted mean Δ (95% CI)	0 (ref)	−1.33 (−3.12, 0.45)	−1.62 (−3.73, 0.50)	−1.46 (−3.83, 0.91)	0.37
Adjusted mean Δ^2^ (95% CI)	0 (ref)	−1.17 (−2.93, 0.59)	−0.91 (−2.98, 1.16)	−1.23 (−3.54, 1.07)	0.56

1 oz = 28.35 g. BMI: body mass index; CI: confidence interval; HDL: high-density lipoproteins; HOMA-IR: homeostatic model assessment of insulin resistance; LDL: low-density lipoproteins; MetS: Metabolic Syndrome; RGM: ratio of geometric means; RR: risk ratio; TG: triglycerides. ^1^ *p*-value testing the null hypothesis that there is no difference between categories of intake. ^2^ Mixed linear regression adjusted for parental sociodemographic status, maternal age, parity, maternal prepregnancy BMI, maternal smoking in pregnancy, maternal physical activity in pregnancy, energy intake; and offspring age and sex.

**Table 3:** The association between consistent fish intake in gestational week 12 and 30 and offspring metabolic parameters at age 9–16 years in GDM-exposed mother-offspring dyads.

Categories of Consistent Fish Intake						
Metabolic measures in the offspring	>2 times/week	1–2 times/week	Weekly	Monthly	Never	*p* ^1^
	*n* = 29 (16%)	*n* = 41 (22%)	*n* = 68 (37%)	*n* = 39 (21%)	*n* = 8 (4%)	
BMI, kg/m^2^ (*n* = 143)						
Unadjusted RGM (95% CI)	1 (ref)	1.08 (0.98, 1.20)	1.09 (0.99, 1.20)	1.03 (0.92, 1.14)	1.22 (1.02, 1.46)	0.11
Adjusted RGM ^2^ (95% CI)	1 (ref)	1.07 (0.96, 1.20)	1.06 (0.96, 1.16)	0.98 (0.88, 1.08)	1.28 (1.06, 1.55)	0.02
Waist circumference, cm (*n* = 143)						
Unadjusted RGM (95% CI)	1 (ref)	1.06 (0.98, 1.15)	1.05 (0.98, 1.13)	1.01 (0.93, 1.09)	1.21 (1.06, 1.39)	0.05
Adjusted RGM ^2^ (95% CI)	1 (ref)	1.04 (0.96, 1.14)	1.02 (0.95, 1.11)	0.97 (0.90, 1.05)	1.22 (1.05, 1.40)	0.02
Total fat mass, % (*n* = 44)						
Unadjusted RGM (95% CI)	1 (ref)	1.09 (0.86, 1.39)	1.08 (0.86, 1.36)	0.99 (0.74, 1.31)	1.26 (0.82, 1.93)	0.75
Adjusted RGM ^2^ (95% CI)	1 (ref)	1.16 (0.79, 1.72)	1.06 (0.77, 1.48)	0.98 (0.66, 1.45)	1.57 (0.51, 4.85)	0.76
Abdominal fat mass, % (*n* = 44)						
Unadjusted RGM (95% CI)	1 (ref)	1.30 (0.84, 2.03)	1.16 (0.76, 1.79)	1.01 (0.59, 1.72)	1.62 (0.73, 3.60)	0.61
Adjusted RGM ^2^ (95% CI)	1 (ref)	1.39 (0.65, 2.97)	1.14 (0.60, 2.16)	0.96 (0.44, 2.12)	2.44 (0.27, 22.20)	0.74
Total cholesterol, mmol/L (*n* = 130)						
Unadjusted mean Δ (95% CI)	0 (ref)	0.14 (−0.29, 0.57)	0.20 (−0.20, 0.60)	−0.06 (−0.49, 0.38)	0.14 (−0.59, 0.87)	0.61
Adjusted mean Δ^2^ (95% CI)	0 (ref)	0.24 (−0.24, 0.72)	0.25 (−0.20, 0.70)	−0.01 (−0.48, 0.46)	0.21 (−0.64, 1.05)	0.56
LDL, mmol/L (*n* = 130)						
Unadjusted mean Δ (95% CI)	0 (ref)	0.15 (−0.26, 0.57)	0.17 (−0.22, 0.56)	0.02 (−0.40, 0.44)	0.08 (−0.63, 0.79)	0.85
Adjusted mean Δ^2^ (95% CI)	0 (ref)	0.16 (−0.31, 0.63)	0.19 (−0.26, 0.63)	0.01 (−0.45, 0.48)	0.16 (−0.68, 0.99)	0.85
HDL, mmol/L (*n* = 130)						
Unadjusted mean Δ (95% CI)	0 (ref)	−0.15 (−0.37, 0.07)	−0.01 (−0.21, 0.20)	−0.15 (−0.37, 0.08)	−0.02 (−0.40, 0.35)	0.34
Adjusted mean Δ^2^ (95% CI)	0 (ref)	−0.09 (−0.32, 0.15)	0.01 (−0.21, 0.24)	−0.11 (−0.34, 0.12)	−0.06 (−0.48, 0.36)	0.62
TG, mmol/L (*n* = 130)						
Unadjusted RGM (95% CI)	1 (ref)	1.17 (0.90, 1.54)	1.21 (0.94, 1.57)	1.21 (0.92, 1.60)	1.72 (1.08, 2.72)	0.22
Adjusted RGM ^2^ (95% CI)	1 (ref)	1.34 (0.98, 1.80)	1.31 (0.99, 1.75)	1.26 (0.94, 1.70)	1.77 (1.03, 3.03)	0.20
HOMA-IR (*n* = 124)						
Unadjusted RGM (95% CI)	1 (ref)	1.22 (0.88, 1.68)	1.52 (1.13, 2.08)	1.15 (0.82, 1.60)	2.23 (1.30, 3.82)	0.01
Adjusted RGM ^2^ (95% CI)	1 (ref)	1.20 (0.84, 1.72)	1.54 (1.09, 2.14)	1.07 (0.76, 1.51)	2.16 (1.17, 3.97)	0.01
MetS ***z*** score, SD (*n* = 123)						
Unadjusted mean Δ (95% CI)	0 (ref)	4.29 (−0.60, 9.17)	6.24 (1.66, 10.8)	2.23 (−2.74, 7.20)	12.47 (4.34, 20.60)	0.01
Adjusted mean Δ^2^ (95% CI)	0 (ref)	4.23 (−0.85, 9.30)	5.94 (1.13, 10.75)	1.04 (−3.91, 5.99)	12.91 (4.07, 21.75)	0.01

BMI: body mass index; CI: confidence interval; HDL: high-density lipoproteins; HOMA-IR: homeostatic model assessment of insulin resistance; LDL: low-density lipoproteins; MetS: Metabolic Syndrome; RGM: ratio of geometric means; RR: risk ratio, TG: triglycerides. ^1^ *p*-value testing the null hypothesis that there is no difference between categories of intake. ^2^ Mixed linear regression adjusted for parental sociodemographic status, maternal age, parity, maternal prepregnancy BMI, maternal smoking in pregnancy, maternal physical activity in pregnancy, energy intake; and offspring age and sex.

## Supplementary Materials

The following are available online at http://www.mdpi.com/2072-6643/10/10/1534/s1, Table S1: Definitions used to classify women with gestational diabetes mellitus, Table S2: The association between intake of total seafood intake in mid-pregnancy and offspring metabolic parameters at age 9–16 years in control mother-offspring dyads, Table S3: The association between consistent fish intake in gestational week 12 and 30 and offspring metabolic parameters at age 9–16 years in control mother-offspring dyads, Table S4: The multivariable association between intake of lean fish intake in mid-pregnancy and offspring metabolic parameters at age 9–16 years, Table S5: The multivariable association between intake of oily fish intake in mid-pregnancy and offspring metabolic parameters at age 9–16 years, Table S6: The multivariable association between intake of marine n-3 LCPUFA from diet in mid-pregnancy and offspring metabolic parameters at age 9–16 years.
