# Supplementary material for: Fish Intake in Pregnancy and Offspring Metabolic Parameters at Age 9–16—Does Gestational Diabetes Modify the Risk?
Source: Nutrients. 2018 Oct 17;10(10):1534. doi: 10.3390/nu10101534 (PMC6213471; doi:10.3390/nu10101534)
Supplement: Supplementary file 1 [file nutrients-10-01534-s001.pdf]

Table S1. Definitions used to classify women with gestational diabetes mellitus

**Test**	**Cut-off points**	**Source**
75 g 3 hours OGTT	>=2 values +3 SDs above the mean for venous plasma glucose. The mean +3SD were:<br>6.2 mmol/L at 0 minutes<br>10.9 mmol/L at 30 minutes<br>11.1 mmol/L at 60 minutes<br>9.2 mmol/L at 90 minutes<br>8.9 mmol/L at 120 minutes<br>8.2 mmol/L at 150 minutes<br>7.3 mmol/L at 180 minutes.<br><br>Borderline glucose tolerance:<br>2 values +2 SDs above the mean for venous plasma glucose:<br>above 5.8 mmol/L at 0 minutes<br>9.6 mmol/L at 30 minutes<br>9.5 mmol/L at 60 minutes<br>8.0 mmol/L at 90 minutes<br>7.7 mmol/L at 120 minutes<br>7.0 mmol/L at 150 minutes<br>6.2 mmol/L at 180 minutes	Curve based on a group of 40 Danish healthy, nonobese, nonpregnant women without a family history of diabetes
75 g 2 hours OGTT	Fasting glucose >7.0 mmol/L, or<br>2 h glucose >7.8 mmol/L	World Health Organization

Table S2. The association between intake of total seafood in gestational week 25 intake and offspring metabolic parameters at age 9-16 years in *control* mother-offspring dyads

	Categories of total seafood intake				
**Metabolic measures in the offspring**	0-0.5 oz/day n=124 (25%)	>0.5-1 oz/day n=167 (34%)	1-1.5 oz/day n=111 (23%)	>1.5 oz/day n=85 (17%)	P[1]
**BMI, kg/m² (n=480)**					
Unadjusted RGM (95% CI)	1 (ref)	0.99 (0.95, 1.02)	1.00 (0.96, 1.04)	0.97 (0.93, 1.01)	0.46
Adjusted RGM[2] (95% CI)	1 (ref)	0.99 (0.96, 1.02)	0.99 (0.96, 1.03)	0.99 (0.96, 1.03)	0.70
**Waist circumference, cm (n=481)**					
Unadjusted RGM (95% CI)	1 (ref)	1.00 (0.97, 1.02)	1.01 (0.98, 1.03)	0.99 (0.95, 1.02)	0.74
Adjusted RGM[2] (95% CI)	1 (ref)	1.00 (0.98, 1.02)	1.00 (0.97, 1.02)	1.00 (0.97, 1.03)	0.99
**Total fat mass, % (n=355)**					
Unadjusted RGM (95% CI)	1 (ref)	0.96 (0.89, 1.03)	0.90 (0.84, 0.98)	0.94 (0.86, 1.03)	0.09
Adjusted RGM[2] (95% CI)	1 (ref)	0.95 (0.90, 1.02)	0.91 (0.85, 0.98)	0.94 (0.87, 1.03)	0.12
**Abdominal fat mass, % (n=355)**					
Unadjusted RGM (95% CI)	1 (ref)	0.92 (0.81, 1.05)	0.85 (0.74, 0.98)	0.91 (0.79, 1.07)	0.18
Adjusted RGM[2] (95% CI)	1 (ref)	0.91 (0.81, 1.03)	0.87 (0.76, 0.99)	0.93 (0.81, 1.08)	0.22
**Total cholesterol, mmol/L (n=428)**					
Unadjusted meanΔ (95% CI)	0 (ref)	-0.15 (-0.33, 0.02)	-0.22 (-0.41, -0.03)	-0.05 (-0.26, 0.17)	0.10
Adjusted meanΔ[2] (95% CI)	0 (ref)	-0.18 (-0.37, 0.00)	-0.23 (-0.42, -0.03)	-0.08 (-0.30, 0.14)	0.09
**LDL, mmol/L (n=428)**					
Unadjusted meanΔ (95% CI)	0 (ref)	-0.15 (-0.30, 0.01)	-0.20 (-0.37, -0.03)	-0.15 (-0.33, 0.04)	0.10
Adjusted meanΔ[2] (95% CI)	0 (ref)	-0.16 (-0.31, 0.00)	-0.18 (-0.35, -0.01)	-0.18 (-0.37, 0.02)	0.13
**HDL, mmol/L (n=428)**					
Unadjusted meanΔ (95% CI)	0 (ref)	0.05 (-0.05, 0.14)	0.01 (-0.09, 0.11)	0.12 (0.01, 0.24)	0.15
Adjusted meanΔ[2] (95% CI)	0 (ref)	0.03 (-0.07, 0.12)	0.00 (-0.10, 0.10)	0.13 (0.01, 0.24)	0.11
**TG, mmol/L (n=428)**					

Unadjusted RGM (95% CI)	1 (ref)	1.00 (0.90, 1.09)	0.95 (0.86, 1.05)	0.97 (0.87, 1.09)	0.76
Adjusted RGM[2] (95% CI)	1 (ref)	0.99 (0.90, 1.08)	0.96 (0.86, 1.06)	0.99 (0.89, 1.12)	0.81
**HOMA-IR (n=409)**					
Unadjusted RGM (95% CI)	1 (ref)	1.02 (0.90, 1.14)	1.01 (0.89, 1.15)	1.02 (0.88, 1.17)	0.99
Adjusted RGM[2] (95% CI)	1 (ref)	1.04 (0.93, 1.17)	1.03 (0.91, 1.17)	1.06 (0.92, 1.22)	0.82
**Metabolic Syndrome *z* score, SD (n=408)**					
Unadjusted meanΔ (95% CI)	0 (ref)	-0.36 (-1.30, 0.58)	-0.19 (-1.21, 0.84)	-0.86 (-1.99, 0.28)	0.50
Adjusted meanΔ[2] (95% CI)	0 (ref)	-0.04 (-0.88, 0.80)	0.01 (-0.92, 0.94)	-0.16 (-1.20, 0.87)	0.99

1 oz = 28.35 g

RGM: ratio of geometric means; RR: risk ratio

[1]P-value testing the null hypothesis that there is no difference between categories of intake.

[2]Mixed linear regression adjusted for parental sociodemographic status, maternal age, parity, maternal prepregnancy BMI, maternal smoking in pregnancy, maternal physical activity in pregnancy, energy intake; and offspring age and sex.

Table S3. The association between consistent fish intake in gestational week 12 and 30 and offspring metabolic parameters at age 9-16 years in *control* mother-offspring dyads

	Categories of consistent fish intake					
**Metabolic measures in the offspring**	>2 times/week n=32 (14%)	1-2 times/week n=70 (30%)	Weekly n=78 (34%)	Monthly n=47 (20%)	Never n=5 (2%)	P[1]
**BMI, kg/m² (n=184)**						
Unadjusted RGM (95% CI)	1 (ref)	0.98 (0.91, 1.04)	0.99 (0.92, 1.05)	0.97 (0.90, 1.04)	1.02 (0.84, 1.23)	0.90
Adjusted RGM[2] (95% CI)	1 (ref)	0.98 (0.93, 1.04)	0.98 (0.93, 1.04)	0.97 (0.91, 1.03)	0.99 (0.84, 1.15)	0.94
**Waist circumference, cm (n=185)**						
Unadjusted RGM (95% CI)	1 (ref)	0.98 (0.93, 1.04)	0.99 (0.93, 1.04)	0.97 (0.91, 1.03)	0.99 (0.85, 1.15)	0.90
Adjusted RGM[2] (95% CI)	1 (ref)	0.99 (0.94, 1.03)	0.99 (0.94, 1.03)	0.98 (0.93, 1.03)	0.96 (0.84, 1.09)	0.88
**Total fat mass, % (n=133)**						
Unadjusted RGM (95% CI)	1 (ref)	1.06 (0.92, 1.23)	1.03 (0.89, 1.20)	1.04 (0.89, 1.21)	1.12 (0.63, 1.97)	0.93
Adjusted RGM[2] (95% CI)	1 (ref)	0.98 (0.87, 1.12)	0.99 (0.87, 1.14)	0.99 (0.85, 1.14)	1.01 (0.60, 1.70)	0.99
**Abdominal fat mass, % (n=133)**						
Unadjusted RGM (95% CI)	1 (ref)	1.03 (0.80, 1.32)	0.98 (0.76, 1.27)	0.98 (0.74, 1.28)	1.39 (0.51, 3.82)	0.95
Adjusted RGM[2] (95% CI)	1 (ref)	0.90 (0.71, 1.14)	0.90 (0.70, 1.15)	0.93 (0.72, 1.22)	1.05 (0.41, 2.69)	0.90
**Total cholesterol, mmol/L (n=170)**						
Unadjusted meanΔ (95% CI)	0 (ref)	0.05 (-0.29, 0.39)	-0.01 (-0.35, 0.33)	0.12 (-0.24, 0.48)	-0.19 (-1.18, 0.81)	0.89
Adjusted meanΔ[2] (95% CI)	0 (ref)	0.06 (-0.30, 0.41)	-0.01 (-0.37, 0.35)	0.17 (-0.21, 0.56)	-0.13 (-1.19, 0.93)	0.76
**LDL, mmol/L (n=170)**						
Unadjusted meanΔ (95% CI)	0 (ref)	0.01 (-0.28, 0.30)	-0.03 (-0.33, 0.26)	0.08 (-0.23, 0.40)	-0.09 (-0.94, 0.75)	0.92
Adjusted meanΔ[2] (95% CI)	0 (ref)	-0.01 (-0.32, 0.29)	-0.03 (-0.34, 0.28)	0.07 (-0.25, 0.39)	-0.10 (-1.02, 0.81)	0.92
**HDL, mmol/L (n=170)**						
Unadjusted meanΔ (95% CI)	0 (ref)	0.11 (-0.08, 0.30)	0.07 (-0.13, 0.26)	-0.07 (-0.27, 0.14)	-0.20 (-0.74, 0.35)	0.22
Adjusted meanΔ[2] (95% CI)	0 (ref)	0.13 (-0.07, 0.33)	0.09 (-0.11, 0.29)	-0.06 (-0.28, 0.16)	-0.15 (-0.73, 0.43)	0.22

**TG, mmol/L (n=170)**						
Unadjusted RGM (95% CI)	1 (ref)	1.04 (0.88, 1.25)	1.02 (0.85, 1.21)	1.15 (0.95, 1.38)	1.49 (0.90, 2.51)	0.24
Adjusted RGM[2] (95% CI)	1 (ref)	1.05 (0.89, 1.26)	1.06 (0.89, 1.27)	1.19 (0.98, 1.43)	1.27 (0.76, 2.16)	0.36
**HOMA-IR (n=161)**						
Unadjusted RGM (95% CI)	1 (ref)	0.95 (0.75, 1.20)	0.95 (0.75, 1.20)	0.99 (0.76, 1.27)	1.09 (0.58, 2.07)	0.97
Adjusted RGM[2] (95% CI)	1 (ref)	0.95 (0.76, 1.19)	0.99 (0.79, 1.25)	0.99 (0.76, 1.28)	0.85 (0.46, 1.58)	0.96
**Metabolic Syndrome *z* score, SD (n=160)**						
Unadjusted meanΔ (95% CI)	0 (ref)	-0.29 (-2.23, 1.65)	0.04 (-1.90, 1.99)	0.88 (-1.21, 2.98)	2.06 (-3.34, 7.46)	0.62
Adjusted meanΔ[2] (95% CI)	0 (ref)	-0.18 (-1.98, 1.61)	0.14 (-1.68, 1.97)	1.08 (-0.95, 3.10)	0.45 (-4.49, 5.39)	0.63

RGM: ratio of geometric means; RR: risk ratio

[1]P-value testing the null hypothesis that there is no difference between categories of intake.

[2]Mixed linear regression adjusted for parental sociodemographic status, maternal age, parity, maternal prepregnancy BMI, maternal smoking in pregnancy, maternal physical activity in pregnancy, energy intake; and offspring age and sex.

Table S4. The multivariable[1] association between intake of lean fish intake in gestational week 25 and offspring metabolic parameters at age 9-16 years

	Categories of lean fish intake			
**Metabolic measures in the offspring**	0 oz/day n=162 (17%)	>0-0.5 oz/day n=587 (63%)	>0.5 oz/day n=181 (19%)	P[2]
**BMI, kg/m^2 (n=920)**				
GDM offspring RGM	1 (ref)	0.95 (0.92, 0.99)	0.98 (0.93, 1.03)	0.03
Control offspring RGM	1 (ref)	0.99 (0.95, 1.02)	0.99 (0.95, 1.03)	0.76
**Waist circumference, cm (n=919)**				
GDM offspring RGM	1 (ref)	0.96 (0.93, 0.99)	0.98 (0.94, 1.01)	0.02
Control offspring RGM	1 (ref)	0.99 (0.97, 1.02)	1.00 (0.96, 1.03)	0.92
**Total fat mass, % (n=506)**				
GDM offspring RGM	1 (ref)	1.02 (0.91, 1.13)	0.95 (0.82, 1.09)	0.56
Control offspring RGM	1 (ref)	0.89 (0.82, 0.96)	0.89 (0.80, 0.97)	0.01
**Abdominal fat mass, % (n=506)**				
GDM offspring RGM	1 (ref)	1.04 (0.85, 1.27)	0.92 (0.70, 1.21)	0.60
Control offspring RGM	1 (ref)	0.82 (0.70, 0.94)	0.84 (0.70, 0.99)	0.03
**Total cholesterol, mmol/L (n=809)**				
GDM offspring meanΔ	0 (ref)	-0.08 (-0.25, 0.10)	0.03 (-0.19, 0.25)	0.46
Control offspring meanΔ	0 (ref)	0.13 (-0.09, 0.35)	0.08 (-0.18, 0.34)	0.49
**LDL, mmol/L (n=809)**				
GDM offspring meanΔ	0 (ref)	-0.01 (-0.18, 0.15)	0.02 (-0.19, 0.22)	0.95
Control offspring meanΔ	0 (ref)	0.09 (-0.11, 0.28)	0.01 (-0.21, 0.24)	0.48
**HDL, mmol/L (n=809)**				
GDM offspring meanΔ	0 (ref)	-0.09 (-0.18, 0.01)	0.02 (-0.09, 0.14)	0.04
Control offspring meanΔ	0 (ref)	-0.01 (-0.13, 0.10)	0.04 (-0.09, 0.18)	0.50
**TG, mmol/L (n=809)**				

GDM offspring RGM	1 (ref)	1.01 (0.90, 1.12)	0.98 (0.86, 1.13)	0.92
Control offspring RGM	1 (ref)	1.09 (0.98, 1.22)	1.07 (0.94, 1.22)	0.31
**HOMA-IR (n=770)**				
GDM offspring RGM	1 (ref)	0.95 (0.83, 1.11)	0.96 (0.80, 1.15)	0.83
Control offspring RGM	1 (ref)	1.13 (0.98, 1.30)	1.22 (1.04, 1.43)	0.05
**Metabolic Syndrome *z* score, SD (n=767)**				
GDM offspring meanΔ	0 (ref)	-0.88 (-2.77, 1.00)	-0.79 (-3.12, 1.55)	0.65
Control offspring meanΔ	0 (ref)	0.70 (-0.32, 1.73)	0.79 (-0.42, 1.99)	0.37

1 oz = 28.35 g

RGM: ratio of geometric means; RR: risk ratio

[1]Mixed linear regression adjusted for parental sociodemographic status, maternal age, parity, maternal prepregnancy BMI, maternal smoking in pregnancy, maternal physical activity in pregnancy, energy intake; and offspring age and sex.

[2]P-value testing the null hypothesis that there is no difference between categories of intake.

Table S5. The multivariable[1] association between intake of oily fish intake in gestational week 25 and offspring metabolic parameters at age 9-16 years

	Categories of oily fish intake			
**Metabolic measures in the offspring**	0 oz/day n=447 (48%)	>0-0.25 oz/day n=288 (31%)	>0.25 oz/day n=195 (21%)	P[2]
**BMI, kg/m² (n=920)**				
GDM offspring RGM	1 (ref)	0.97 (0.93, 1.00)	0.98 (0.94, 1.02)	0.19
Control offspring RGM	1 (ref)	0.99 (0.96, 1.02)	0.99 (0.96, 1.02)	0.66
**Waist circumference, cm (n=919)**				
GDM offspring RGM	1 (ref)	0.97 (0.95, 1.00)	1.00 (0.96, 1.03)	0.18
Control offspring RGM	1 (ref)	1.00 (0.98, 1.02)	1.00 (0.98, 1.02)	0.95
**Total fat mass, % (n=506)**				
GDM offspring RGM	1 (ref)	1.04 (0.93, 1.16)	1.00 (0.90, 1.12)	0.76
Control offspring RGM	1 (ref)	1.00 (0.94, 1.06)	0.97 (0.91, 1.03)	0.61
**Abdominal fat mass, % (n=506)**				
GDM offspring RGM	1 (ref)	1.07 (0.87, 1.32)	1.01 (0.83, 1.22)	0.79
Control offspring RGM	1 (ref)	1.00 (0.90, 1.12)	0.97 (0.87, 1.09)	0.85
**Total cholesterol, mmol/L (n=809)**				
GDM offspring meanΔ	0 (ref)	-0.01 (-0.17, 0.16)	0.08 (-0.11, 0.27)	0.69
Control offspring meanΔ	0 (ref)	0.03 (-0.13, 0.19)	0.05 (-0.13, 0.23)	0.86
**LDL, mmol/L (n=809)**				
GDM offspring meanΔ	0 (ref)	-0.04 (-0.20, 0.11)	0.05 (-0.13, 0.22)	0.66
Control offspring meanΔ	0 (ref)	0.02 (-0.12, 0.16)	-0.03 (-0.19, 0.12)	0.78
**HDL, mmol/L (n=809)**				
GDM offspring meanΔ	0 (ref)	0.01 (-0.08, 0.10)	0.05 (-0.05, 0.15)	0.61
Control offspring meanΔ	0 (ref)	0.04 (-0.04, 0.13)	0.07 (-0.02, 0.16)	0.30
**TG, mmol/L (n=809)**				

GDM offspring RGM	1 (ref)	0.99 (0.90, 1.08)	0.97 (0.87, 1.09)	0.88
Control offspring RGM	1 (ref)	1.03 (0.95, 1.12)	1.00 (0.91, 1.09)	0.64
**HOMA-IR (n=770)**				
GDM offspring RGM	1 (ref)	1.00 (0.87, 1.14)	0.95 (0.82, 1.12)	0.82
Control offspring RGM	1 (ref)	1.02 (0.92, 1.13)	0.97 (0.86, 1.08)	0.67
**Metabolic Syndrome *z* score, SD (n=767)**				
GDM offspring meanΔ	0 (ref)	-0.70 (-2.38, 0.98)	-0.86 (-2.87, 1.14)	0.58
Control offspring meanΔ	0 (ref)	0.29 (-0.45, 1.04)	-0.34 (-1.18, 0.49)	0.32

1 oz = 28.35 g

RGM: ratio of geometric means; RR: risk ratio

[1]Mixed linear regression adjusted for parental sociodemographic status, maternal age, parity, maternal prepregnancy BMI, maternal smoking in pregnancy, maternal physical activity in pregnancy, energy intake; and offspring age and sex.

[2]P-value testing the null hypothesis that there is no difference between categories of intake.

Table S6. The multivariable[1] association between intake of marine n-3 LCPUFA from diet in gestational week 25 and offspring metabolic parameters at age 9-16 years

		Quartiles of marine n-3 LCPUFA intake				
**Metabolic measures in the offspring**	Continuous	1	2	3	4	P[2]
**BMI, kg/m[2] (n=920)**						
GDM offspring	0.98 (0.94, 1.03)	1 (ref)	1.00 (0.96, 1.04)	0.99 (0.95, 1.03)	0.98 (0.94, 1.02)	0.72
Control offspring	1.01 (0.98, 1.05)	1 (ref)	0.99 (0.96, 1.02)	1.01 (0.97, 1.04)	1.00 (0.97, 1.03)	0.80
**Waist circumference, cm (n=919)**						
GDM offspring	1.00 (0.96, 1.04)	1 (ref)	1.01 (0.97, 1.04)	0.99 (0.96, 1.02)	0.99 (0.95, 1.02)	0.64
Control offspring	1.02 (0.99, 1.05)	1 (ref)	1.00 (0.98, 1.03)	1.01 (0.98, 1.03)	1.00 (0.98, 1.03)	0.89
**Total fat mass, % (n=506)**						
GDM offspring	1.08 (0.95, 1.22)	1 (ref)	1.04 (0.91, 1.20)	1.08 (0.95, 1.22)	1.11 (0.97, 1.25)	0.44
Control offspring	0.97 (0.90, 1.05)	1 (ref)	1.00 (0.93, 1.08)	1.01 (0.94, 1.08)	0.98 (0.91, 1.05)	0.81
**Abdominal fat mass, % (n=506)**						
GDM offspring	1.12 (0.89, 1.40)	1 (ref)	1.06 (0.83, 1.36)	1.19 (0.93, 1.49)	1.17 (0.93, 1.48)	0.42
Control offspring	0.98 (0.86, 1.13)	1 (ref)	0.98 (0.86, 1.13)	1.03 (0.90, 1.17)	0.98 (0.86, 1.11)	0.86
**Total cholesterol, mmol/L (n=809)**						
GDM offspring	-0.12 (-0.34, 0.10)	0 (ref)	0.11 (-0.09, 0.31)	0.00 (-0.19, 0.20)	-0.04 (-0.24, 0.16)	0.50
Control offspring	0.00 (-0.22, 0.22)	0 (ref)	0.08 (-0.12, 0.29)	-0.08 (-0.28, 0.12)	0.01 (-0.19, 0.21)	0.46
**LDL, mmol/L (n=809)**						
GDM offspring	-0.12 (-0.32, 0.08)	0 (ref)	0.12 (-0.06, 0.30)	0.00 (-0.18, 0.19)	-0.01 (-0.19, 0.18)	0.46
Control offspring	-0.08 (-0.27, 0.10)	0 (ref)	0.06 (-0.11, 0.24)	-0.05 (-0.22, 0.12)	-0.06 (-0.24, 0.11)	0.41
**HDL, mmol/L (n=809)**						
GDM offspring	0.00 (-0.12, 0.11)	0 (ref)	-0.05 (-0.15, 0.05)	-0.01 (-0.11, 0.09)	-0.03 (-0.14, 0.07)	0.77
Control offspring	0.08 (-0.04, 0.19)	0 (ref)	0.06 (-0.04, 0.17)	0.04 (-0.07, 0.14)	0.08 (-0.02, 0.18)	0.46
**TG, mmol/L (n=809)**						

GDM offspring	0.89 (0.78, 1.01)	1 (ref)	1.02 (0.91, 1.15)	0.95 (0.85, 1.07)	0.93 (0.83, 1.04)	0.35
Control offspring	0.97 (0.87, 1.08)	1 (ref)	1.05 (0.94, 1.16)	1.02 (0.92, 1.13)	0.97 (0.88, 1.07)	0.49
**HOMA-IR (n=770)**						
GDM offspring	0.91 (0.76, 1.09)	1 (ref)	0.96 (0.82, 1.12)	0.91 (0.78, 1.06)	0.90 (0.76, 1.05)	0.48
Control offspring	0.95 (0.84, 1.09)	1 (ref)	1.01 (0.89, 1.14)	1.08 (0.96, 1.23)	0.97 (0.85, 1.09)	0.24
**Metabolic Syndrome score (n=767)**						
GDM offspring	-1.14 (-3.45, 1.17)	0 (ref)	-0.55 (-2.55, 1.45)	-1.41 (-3.34, 0.52)	-1.71 (-3.77, 0.36)	0.31
Control offspring	0.05 (-0.95, 1.06)	0 (ref)	-0.08 (-1.03, 0.87)	0.14 (-0.79, 1.06)	-0.26 (-1.19, 0.67)	0.84

RGM: ratio of geometric means; RR: risk ratio

[1]Mixed linear regression adjusted for parental sociodemographic status, maternal age, parity, maternal prepregnancy BMI, maternal smoking in pregnancy, maternal physical activity in pregnancy, energy intake; and offspring age and sex.

[2]P-value testing the null hypothesis that there is no difference between categories of intake.
